# Major Burden of Severe Anemia from Non-Falciparum Malaria Species in Southern Papua: A Hospital-Based Surveillance Study

**DOI:** 10.1371/journal.pmed.1001575

**Published:** 2013-12-17

**Authors:** Nicholas M. Douglas, Daniel A. Lampah, Enny Kenangalem, Julie A. Simpson, Jeanne R. Poespoprodjo, Paulus Sugiarto, Nicholas M. Anstey, Ric N. Price

**Affiliations:** 1 Global Health Division, Menzies School of Health Research and Charles Darwin University, Darwin, Australia; 2 Centre for Tropical Medicine, Nuffield Department of Clinical Medicine, University of Oxford, United Kingdom; 3 Timika Malaria Research Programme, Papuan Health and Community Development Foundation, Timika, Papua, Indonesia; 4 Mimika District Health Authority, Timika, Papua, Indonesia; 5 Centre for Molecular, Environmental, Genetic and Analytic Epidemiology, Melbourne School of Population Health, University of Melbourne, Victoria, Australia; 6 Rumah Sakit Mitra Masyarakat, Timika, Papua, Indonesia; 7 Division of Medicine, Royal Darwin Hospital, Darwin, Australia; University of Copenhagen and Rigshospitalet, Denmark

## Abstract

**Background:**

The burden of anemia attributable to non-falciparum malarias in regions with *Plasmodium* co-endemicity is poorly documented. We compared the hematological profile of patients with and without malaria in southern Papua, Indonesia.

**Methods and Findings:**

Clinical and laboratory data were linked for all patients presenting to a referral hospital between April 2004 and December 2012. Data were available on patient demographics, malaria diagnosis, hemoglobin concentration, and clinical outcome, but other potential causes of anemia could not be identified reliably. Of 922,120 patient episodes (837,989 as outpatients and 84,131 as inpatients), a total of 219,845 (23.8%) were associated with a hemoglobin measurement, of whom 67,696 (30.8%) had malaria. Patients with *P. malariae* infection had the lowest hemoglobin concentration (*n* = 1,608, mean = 8.93 [95% CI 8.81–9.06]), followed by those with mixed species infections (*n* = 8,645, mean = 9.22 [95% CI 9.16–9.28]), *P. falciparum* (*n* = 37,554, mean = 9.47 [95% CI 9.44–9.50]), and *P. vivax* (*n* = 19,858, mean = 9.53 [95% CI 9.49–9.57]); *p*-value for all comparisons <0.001. Severe anemia (hemoglobin <5 g/dl) was present in 8,151 (3.7%) patients. Compared to patients without malaria, those with mixed *Plasmodium* infection were at greatest risk of severe anemia (adjusted odds ratio [AOR] 3.25 [95% CI 2.99–3.54]); AORs for severe anaemia associated with *P. falciparum*, *P. vivax*, and *P. malariae* were 2.11 (95% CI 2.00–2.23), 1.87 (95% CI 1.74–2.01), and 2.18 (95% CI 1.76–2.67), respectively, *p*<0.001. Overall, 12.2% (95% CI 11.2%–13.3%) of severe anemia was attributable to non-falciparum infections compared with 15.1% (95% CI 13.9%–16.3%) for *P. falciparum* monoinfections. Patients with severe anemia had an increased risk of death (AOR = 5.80 [95% CI 5.17–6.50]; *p*<0.001). Not all patients had a hemoglobin measurement, thus limitations of the study include the potential for selection bias, and possible residual confounding in multivariable analyses.

**Conclusions:**

In Papua *P. vivax* is the dominant cause of severe anemia in early infancy, mixed *P. vivax/P. falciparum* infections are associated with a greater hematological impairment than either species alone, and in adulthood *P. malariae*, although rare, is associated with the lowest hemoglobin concentration. These findings highlight the public health importance of integrated genus-wide malaria control strategies in areas of *Plasmodium* co-endemicity.

*Please see later in the article for the Editors' Summary*

## Abstract 2

Ric Price and colleagues use hospital-based surveillance data to estimate the risk of severe anemia and mortality associated with endemic *Plasmodium* species in southern Papua, Indonesia.

*Please see later in the article for the Editors' Summary*

## Editors' Summary

**Background:**

Malaria—a mosquito-borne parasitic disease—is a global public health problem. Five parasites cause malaria—*Plasmodium falciparum*, *P. vivax*, *P. ovale*, *P. malariae*, and *P. knowlesi*. Of these, *P. vivax* is the commonest and most widely distributed, whereas *P. falciparum* causes the most deaths—about a million every year. All these parasites enter their human host when an infected mosquito takes a blood meal. The parasites migrate to the liver where they replicate and mature into a parasitic form known as merozoites. After 8–9 days, the merozoites are released from the liver cells and invade red blood cells where they replicate rapidly before bursting out and infecting more red blood cells. Malaria's recurring flu-like symptoms are caused by this cyclical increase in parasites in the blood. Malaria needs to be treated promptly with antimalarial drugs to prevent the development of potentially fatal complications. Infections with *P. falciparum* in particular can cause anemia (a reduction in red blood cell numbers) and can damage the brain and other vital organs by blocking the capillaries that supply these organs with blood.

**Why Was This Study Done?:**

It is unclear what proportion of anemia is attributable to non-falciparum malarias in regions of the world where several species of malaria parasite are always present (*Plasmodium* co-endemicity). Public health officials in such regions need to know whether non-falciparum malarias are a major cause of anemia when designing malaria control strategies. If *P. vivax*, for example, is a major cause of anemia in an area where *P. vivax* and *P. falciparum* co-exist, then any malaria control strategies that are implemented need to take into account the biological differences between the parasites. In this hospital-based cohort study, the researchers investigate the burden of severe anemia from the endemic *Plasmodium* species in southern Papua, Indonesia.

**What Did the Researchers Do and Find?:**

The researchers used hospital record numbers to link clinical and laboratory data for patients presenting to a referral hospital in southern Papua over an 8-year period. The hemoglobin level (an indicator of anemia) was measured in about a quarter of hospital presentations (some patients attended the hospital several times). A third of the presentations who had their hemoglobin level determined (67,696 presentations) had clinical malaria. Patients with *P. malariae* infection had the lowest average hemoglobin concentration. Patients with mixed species, *P. falciparum*, and *P. vivax* infections had slightly higher average hemoglobin levels but all these levels were below the normal range for people living in Papua. Among the patients who had their hemoglobin status assessed, 3.7% had severe anemia. After allowing for other factors that alter the risk of anemia (“confounding” factors such as age), patients with mixed *Plasmodium* infection were more than three times as likely to have severe anemia as patients without malaria. Patients with *P. falciparum*, *P. vivax*, or *P. malariae* infections were about twice as likely to have severe anemia as patients without malaria. About 12.2% of severe anemia was attributable to non-falciparum infections, 15.1% was attributable to *P. falciparum* monoinfections, and *P. vivax* was the dominant cause of severe anemia in infancy. Finally, compared to patients without anemia, patients with severe anemia had nearly a 6-fold higher risk of death.

**What Do These Findings Mean?:**

These findings provide a comparative assessment of the pattern of anemia associated with non-falciparum malarias in Papua and an estimate of the public health importance of these malarias. Although the accuracy of these findings may be affected by residual confounding (for example, the researchers did not consider nutritional status when calculating how much malaria infection increases the risk of anemia) and other limitations of the study design, non-falciparum malarias clearly make a major contribution to the burden of anemia in southern Papua. In particular, these findings reveal the large contribution that *P. vivax* makes to severe anemia in infancy, show that the hematological (blood-related) impact of *P. malariae* is most apparent in adulthood, and suggest, in contrast to some previous reports, that mixed *P. vivax*/*P. falciparum* infection is associated with a higher risk of severe anemia than monoinfection with either species. These findings, which need to be confirmed in other settings, highlight the public health importance of implementing integrated malaria control strategies that aim to control all *Plasmodium* species rather than a single species in regions of *Plasmodium* co-endemicity.

**Additional Information:**

Please access these websites via the online version of this summary at http://dx.doi.org/10.1371/journal.pmed.1001575.

- This study is further discussed in a *PLOS Medicine* Perspective by Gosling and Hsiang
- Information is available from the World Health Organization on malaria (in several languages); the 2012 World Malaria Report provides details of the current global malaria situation
- The US Centers for Disease Control and Prevention provide information on malaria (in English and Spanish), including information on different Plasmodium species and a selection of personal stories about malaria
- The Malaria Vaccine Initiative has fact sheets on Plasmodium falciparum malaria and on Plasmodium vivax malaria
- MedlinePlus provides links to additional information on malaria and on anemia (in English and Spanish)
- Information is available from the WorldWide Antimalarial Resistance Network on antimalarial drug resistance for *P. falciparum* and *P. vivax*

## Introduction

Anemia is a common manifestation of *Plasmodium* infection and is responsible for substantial morbidity [1]–[4] as well as direct [5]–[7] and indirect mortality [8]–[11]. Its pathogenesis is incompletely understood. Acute falciparum malaria results in increased removal from the circulation of parasitized and, to a greater extent, non-parasitized red blood cells through a combination of splenic filtration [12], schizont rupture, macrophage phagocytosis [13], complement-mediated hemolysis [14], and increased free radical damage [15],[16]. In more chronic infections, decreased marrow production of functional red blood cells due to the direct inhibitory effects of parasites [17] and cytokines [13],[18] along with dysregulation of erythropoietin and iron metabolism [19] adds to the anemia of ongoing red blood cell loss [16]. Although some of these processes have been described in the non-falciparum human malarias [20]–[23], less is known about the epidemiology and pathogenesis of anemia due to these *Plasmodium* species [24]–[26].

In regions of high *P. falciparum* endemicity, repeated infections from an early age induce robust immunity to clinical disease and a low risk of severe anemia beyond childhood [27]. However, much of the malarious world has low or moderate malarial endemicity. Outside of Africa, *P. falciparum* invariably co-exists with other *Plasmodium* species—the most important of which is *P. vivax*. In these regions, less intense parasite exposure during early life delays the development of immunity and gives rise to the potential for symptomatic and complicated infections at all ages.

Biological differences and interactions between *P. falciparum* and the other *Plasmodium* species complicate analyses of the pattern and public health impact of malarial anemia in co-endemic areas. *Plasmodium vivax* infection has been shown to reduce the risk of anemia secondary to *P. falciparum* malaria, possibly by providing a degree of cross-species immunity [28]–[31]. However, recent population-based studies have revealed a high risk of severe anemia associated with *P. vivax* infection and mixed-species infections suggesting that the hematological impact of the non-falciparum malarias may have been underestimated [30],[32]–[35]. The age-associated changes in risk of anemia in non-falciparum and mixed-species infections and the consequences of anemia caused by these species are largely unknown [26],[33],[34].

In the present study we used clinical and laboratory data from Mitra Masyarakat Hospital in southern Papua, Indonesia, to establish the comparative hematological profiles of patients infected by the different *Plasmodium* species in a co-endemic setting.

## Methods

### Ethical Approval

Ethical approval for this study was obtained from the Health Research Ethics Committees of the University of Gadjah Mada, Indonesia, Menzies School of Health Research, Darwin, Australia, and the Oxford Tropical Centre, Oxford, UK.

### Study Site

Mimika District lies in south-central Papua, the easternmost province of Indonesia. Its geography, climate, and demographics have been described elsewhere [33],[36],[37]. In brief, censuses in 2004 and 2007 estimated the local population to be 130,000 and 170,000 people, respectively, approximately 50% of whom were indigenous Papuans; the remaining 50% being Indonesians from elsewhere in the archipelago [37]. Malaria transmission is limited to lowland areas where it is associated with three mosquito vectors: *Anopheles koliensis*, *An. farauti*, and *An. punctulatus*. The estimated average annual incidence of parasitemia is 876 episodes per 1,000 people, 58% due to *P. falciparum*, 37% due to *P. vivax*, 3% due to mixed infection, and 1.8% due to *P. malariae* [37]. The point prevalence of asexual parasitemia in 2005 was estimated to be 7.5% for *P. falciparum*, 6.4% for *P. vivax*, 1.9% for mixed infection, and 0.6% for *P. malariae* [37].

Until November 2008, Rumah Sakit Mitra Masyarakat (RSMM) was the only referral hospital in the district; since 2008 RSMM treated approximately 80% of patients with malaria attending an inpatient facility in the district. RSMM has 110 beds, a high dependency unit, a 24-hour emergency department, and a busy outpatients department that reviews approximately 300 patients per day, 6 days per week. Treatment is provided free of charge to Papuans and at a cost for non-Papuans.

### Laboratory and Data Collection Procedures

Protocols dictate that all patients presenting to the outpatients department with a fever or symptoms consistent with malaria and all inpatients, regardless of diagnosis, should have a malaria blood film. As a result, reliance on clinical symptoms alone to make the diagnosis of malaria is discouraged and rare. Microbiological diagnosis of malaria is usually based on a thick film examination, though confirmatory thin films and histidine rich protein (HRP2)-based rapid diagnostic tests for *P. falciparum* (Paracheck) are also performed in some cases. In 2004, a random selection of 1,083 positive slides was re-read by an independent expert microscopist with more than 10 years of experience. Concordance was 90% with 1.7% of cases reported as negative on the second reading and 4% of monoinfections reclassified as mixed infections. Complete blood counts are ordered according to clinical indication and are performed using a Coulter Counter (JT Coulter).

Every patient presenting to RSMM for the first time (regardless of department) is assigned a unique hospital record number (HRN). All clinical, laboratory, and pharmacy data from the first and subsequent presentations are linked to this unique HRN. Hospital clerks record basic demographic and administrative information, mortality data, and the diagnoses given by the attending doctor (classified according to the International Classification of Diseases) for each patient presentation to hospital. Ethnicity is recorded as the patient's “Suku” (clan), which is self-reported to the admission clerk on presentation to the hospital. For the purposes of analyses, ethnicity was categorized as Highland Papuan, Lowland Papuan, or non-Papuan on the basis of the location of the clans' village(s). Hematological results are generated by coulter counter (JT Coulter) and collated in a separate laboratory database. Pharmacy data are entered manually into an electronic database by the pharmacist fulfilling the prescription. Since data were gathered from routine hospital surveillance informed consent was not requested from participants, however all records were anonymized to ensure patient confidentiality.

### Data Merging and Statistical Analyses

**Figure 1:**
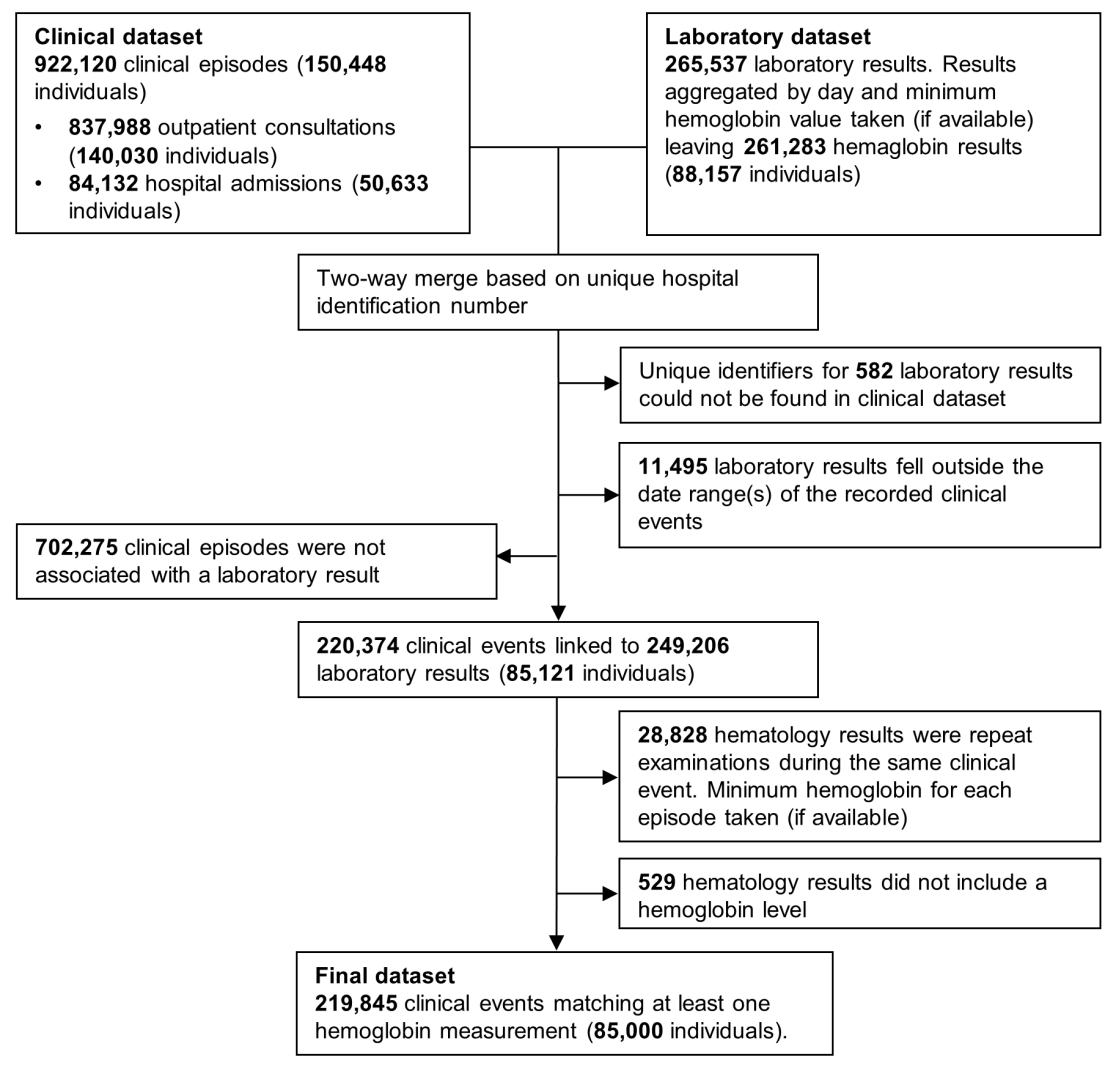
Flow diagram of the merging process.

Clinical data were merged with hematology data by creating all possible pairwise combinations for each HRN. Pairs in which the laboratory record fell between the date of presentation and discharge (the same day for outpatient visitations) were kept and if more than one hemoglobin measurement was available for a single event, the lowest was taken (Figure 1). For the purposes of these analyses, mixed infection was defined as concomitant infection with any combination of *Plasmodium* species.

The primary outcomes in this study were the mean hemoglobin concentration (g/dl) associated with infection by the different *Plasmodium* species with comparison to patients without malaria, presence of severe anemia (hemoglobin less than 5 g/dl), the population attributable fraction (PAF) of severe anemia associated with infection by the different *Plasmodium* species at all ages from infancy through to adulthood, and all-cause mortality. Continuous hemoglobin data were analysed using linear regression and binary anemia data (such as severe anemia and death) were analysed using logistic regression. Since some patients appeared in the database multiple times, robust standard errors were calculated using the Huber-White sandwich estimator.

Univariable analyses were performed for each of the following variables: *Plasmodium* species (negative, *P. falciparum, P. vivax, P. malariae, P. ovale*, or mixed species), sex, self-reported ethnicity (non-Papuan, Highland Papuan, Lowland Papuan), age group (<1 year, 1 to <5 years, 5 to <15 years, ≥15 years), and year of presentation (2004 through to 2012). All of these factors, as well as the interaction between age and *Plasmodium* species, were associated with clinically important differences in hematological status and included in multivariable models. Department (outpatient versus inpatient) and the number of presentations with malaria in the preceding 2 months (0, 1, or 2 or more) were also included in univariable analyses; however, both factors were omitted from multivariable analyses since department was deemed to be a consequence of severe disease rather than a confounder, and the number of preceding malaria episodes would potentially obscure the net hematological effect of *Plasmodium* infection. Fractional polynomials were used to allow for the non-linear relationship between age (as a continuous exposure) and the mean hemoglobin and risk of severe anemia, because they make no assumption regarding the shape of this association [38]. To maintain stability of the fractional polynomial models infants under 1 week of age (*n* = 1114, 0.51%) were excluded since they would not have had *ex utero* exposure to malaria. Adults over 63 years (the 99th percentile) of age (*n* = 2,405, 1.1%) were also excluded.

Adjusted PAFs of severe anemia associated with *Plasmodium* infection were calculated for multiple age groups from infancy through to adulthood from multivariable logistic regression models using the punaf module for STATA, which derives PAFs using the formulae provided in Greenland and Drescher [39]. No allowances were made for within-patient correlation in these analyses since the primary interest was the total hospital workload rather than the hematological status of individuals. Due to very small numbers, patients with *P. ovale* infections were excluded (*n* = 31, 0.01%) from the multivariable models. All statistical analyses were done in STATA version 12.1 (StataCorp).

## Results

**Figure 2:**
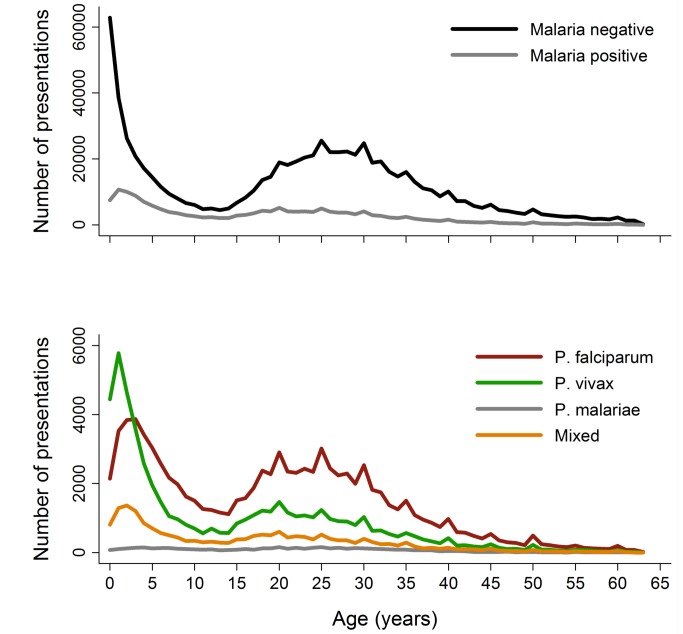
Age distribution of patient presentations to hospital by malaria status (top) and *Plasmodium* species (bottom).

Between April 2004 and December 2012, there were 922,120 patient presentations to RSMM Hospital constituting 837,989 (90.9%) outpatient visitations and 84,131 (9.1%) inpatient admissions (Table 1). The age distribution of all patient presentations combined showed a peak in infancy and a second peak during the late 20 s (Figure 2). Microscopically confirmed malaria was diagnosed in 18.3% (168,525) of patient presentations, with *P. falciparum* accounting for 53.3% of monoinfections, *P. vivax* for 32.3%, *P. malariae* 2.7%, and *P. ovale* 0.06%. Mixed species infections were detected in 19,569 (11.6%) presentations, which in 18,489 (94.5%) cases were mixed *P. falciparum* and *P. vivax* infections.

**Table 1:** Distribution of clinical and laboratory data plus hematological status by clinical and demographic group.

Characteristics	Total Clinical Events	Total Number of Patients	Distribution of Clinical and Laboratory Data				Hematological Status				
			OP Events	OP Events with Hb (%)	IP Events	IP Events with Hb (%)	Mean Hb	SD g/dl	*p*-Value*	Hb<5 g/dl *n* (%)	*p*-Value*
**Species**											
**Negative**	753,595	135,229	695,051	107,436 (15.5)	58,544	44,713 (76.4)	10.51	2.65		3,844 (2.5)	Ref
***P. falciparum***	89,748	44,171	73,392	22,225 (30.3)	16,356	15,329 (93.7)	9.47	2.89		2,444 (6.5)	<0.001
***P. vivax***	54,495	28,841	49,041	14,709 (30)	5,454	5,149 (94.4)	9.53	2.73		1,050 (5.3)	<0.001
**Mixed infection**	19,569	14,206	16,210	5,492 (33.9)	3,359	3,153 (93.9)	9.22	2.86		718 (8.3)	<0.001
***P. malariae***	4,598	4,045	4,190	1,222 (29.2)	408	386 (94.6)	8.93	2.54		95 (5.9)	<0.001
***P. ovale***	115	110	105	22 (21)	10	9 (90)	10.27	2.78		0 (0)	—
**Sex**											
**Male**	416,065	80,186	379,390	71,060 (18.7)	36,675	29,092 (79.3)	10.61	3		3,641 (3.6)	Ref
**Female**	506,055	70,493	458,599	80,046 (17.5)	47,456	39,647 (83.5)	9.82	2.48		4,510 (3.8)	0.2
**Ethnic group**											
**Non-Papuan**	149,017	44,196	136,604	23,357 (17.1)	12,413	9,429 (76)	12.16	2.52	Ref	365 (1.1)	Ref
**Highland Papuan**	644,073	79,443	586,388	107,569 (18.3)	57,685	48,401 (83.9)	9.78	2.66	<0.001	6,933 (4.4)	<0.001
**Lowland Papuan**	127,385	26,413	113,440	19,969 (17.6)	13,945	10,850 (77.8)	10.09	2.57	<0.001	851 (2.8)	<0.001
**Age**											
**<1 year**	70,221	20,068	55,359	13,187 (23.8)	14,862	6,753 (45.4)	9.59	2.36	<0.001	636 (3.2)	<0.001
**1 to <5 years**	139,033	23,596	124,985	29,827 (23.9)	14,048	11,482 (81.7)	9.27	2.31	<0.001	2,094 (5.1)	<0.001
**5 to <15 years**	107,163	23,318	100,319	19,673 (19.6)	6,844	6,306 (92.1)	9.66	2.5	<0.001	1,217 (4.7)	<0.001
**≥15 years**	605,588	101,728	557,250	88,389 (15.9)	48,338	44,170 (91.4)	10.66	2.88	Ref	4,198 (3.2)	Ref
**Number of malaria presentations in last 2 m**											
**0**	799,988	150,448	724,429	128,954 (17.8)	75,559	60,717 (80.4)	10.32	2.77		6,789 (3.6)	
**1**	105,205	30,570	98,054	18,907 (19.3)	7,151	6,695 (93.6)	9.42	2.52		1,110 (4.3)	
**2+**	16,927	6,972	15,506	3,245 (20.9)	1,421	1,327 (93.4)	8.9	2.36		252 (5.5)	
**Year**											
**2004**	62,985	25,864	56,227	8,405 (14.9)	6,758	4,565 (67.5)	9.03	2.64	Ref	925 (7.1)	Ref
**2005**	88,400	30,950	78,398	16,825 (21.5)	10,002	8,615 (86.1)	9.56	2.69	<0.001	1,114 (4.4)	<0.001
**2006**	96,086	34,260	86,288	12,686 (14.7)	9,798	8,292 (84.6)	9.81	2.78	<0.001	1,034 (4.9)	<0.001
**2007**	106,046	36,914	95,272	13,847 (14.5)	10,774	9,176 (85.2)	9.65	2.89	<0.001	1,478 (6.4)	0.01
**2008**	98,074	34,127	88,967	11,282 (12.7)	9,107	7,582 (83.3)	10.31	2.9	<0.001	832 (4.4)	<0.001
**2009**	110,796	34,341	100,756	20,378 (20.2)	10,040	8,285 (82.5)	10.34	2.62	<0.001	778 (2.7)	<0.001
**2010**	112,566	35,041	103,263	22,396 (21.7)	9,303	7,669 (82.4)	10.54	2.61		703 (2.3)	
**2011**	115,683	37,055	106,640	19,483 (18.3)	9,043	7,023 (77.7)	10.7	2.6		559 (2.1)	
**2012**	131,484	41,495	122,178	25,804 (21.1)	9,306	7,532 (80.9)	10.76	2.7		728 (2.2)	
**Total**	922,120	105,448	837,989	151,106 (18.0)	84,131	68,739 (81.7)	10.18	2.76	—	8,151 (3.7)	—

*p*-Values based on univariable linear regression with correction of the variance-covariance matrix for within patient correlation.

OP, outpatient; IP, inpatient; hb, hemoglobin; Ref, reference category.

The absolute number of patient presentations with malaria over the study period peaked during the second year of life but as a proportion of all patient presentations was highest during the early teens (Figure 2). *Plasmodium vivax* infection was the dominant cause of malaria in patients under 3 years of age in both the outpatient and inpatient setting. Thereafter, *P. falciparum* was the most common malaria parasite.

### Availability of Hemoglobin Data

Overall, 219,845 (23.8%) patient presentations (made by 85,000 individuals) were matched with at least one hemoglobin measurement. Of these 85,000 individuals, 40,427 (47.6%) had more than one presentation to hospital; the number of presentations ranging from 1 to 56 (median = 2). Excluding *P. ovale* (which was rare), 30.6% of malaria outpatient presentations were matched with a hemoglobin measurement compared to 15.5% of non-malaria presentations. The corresponding figures for patients admitted to the wards were 93.9% and 76.4%, respectively. Patients who had a hemoglobin measurement were slightly younger than those who did not have a measurement, the median age being 23.0 years versus 24.5 years in those without malaria and 15.9 years versus 18.2 years for those with malaria; *p*<0.001 for both comparisons. Measurement of hemoglobin was also more common in males compared to females (odds ratio = 1.02 [95% CI 1.01–1.03]) and Papuans compared to non-Papuans (odds ratio = 1.13 [95% CI 1.12–1.15]); *p*<0.001 for both comparisons.

The mean of the minimum hemoglobin concentration recorded for each patient presentation was 10.18 g/dl and the maximum 10.34 g/dl. The mean difference between minimum and maximum hemoglobin was greatest for patients with *P. falciparum* (either alone 0.21 g/dl [standard deviation (SD) = 0.82] or mixed 0.24 g/dl [SD = 0.88]) compared to 0.17 g/dl (SD = 0.80) in patients with *P. vivax* and 0.14 g/dl (SD = 0.72) in those with *P. malariae*; *p*<0.001.

In total 13.7% (30,174/219,845) of the patients with a hemoglobin measurement had presented to hospital with malaria within the previous 2 months, the number of malaria episodes ranging from 1 to 6. The risk of recent malaria presentation was greatest in patients presenting with *P. vivax* either alone or mixed (20.5%, 5,840/28,503) compared to those with *P. falciparum* alone (14.6%, 5,493/37,554), *P. malariae* (6%, 97/1,608), or those without malaria (12.3% 18,742/152,149); *p*<0.0001. Recent malaria caused by any species was most common in young children aged 1 to 5 years old with an odds ratio of 2.29 (95% CI 2.21–2.37; *p*<0.0001) compared to adults (see Table 2).

**Table 2:** Univariable and multivariable analyses of the risk factors for presentation with malaria in the previous 2 months (*n* = 219,845).

Characteristics	Univariable Analysis		Multivariable Analysis		
	Crude OR [95% CI]	*p*-Value	AOR [95% CI]	*p*-Value	PAF % [95% CI]
**Species**					
**Negative**	Reference		Reference		
***P. falciparum***	1.48 [1.45–1.51]	<0.001	1.24 [1.21–1.27]	<0.001	2.0 [1.8–2.2]
***P. vivax***	1.90 [1.85–1.95]	<0.001	1.41 [1.37–1.45]	<0.001	2.2 [2.0–2.4]
***P. malariae***	0.49 [0.43–0.55]	<0.001	0.38 [0.34–0.44]	<0.001	<0 [<0–<0]
**Mixed species**	1.93 [1.86–2.01]	<0.001	1.39 [1.33–1.44]	<0.001	0.8 [0.7–0.9]
**Age group**					
**≥15 years**	Reference		Reference		
**<1 year**	0.93 [0.88–0.98]	0.01	0.92 [0.88–0.97]	0.003	<0 [<0–<0]
**1 to <5 years**	2.29 [2.21–2.37]	<0.001	2.09 [2.02–2.16]	<0.001	11.3 [11.1–11.6]
**5 to <15 years**	1.48 [1.42–1.53]	<0.001	1.31 [1.27–1.36]	<0.001	2.8 [2.6–3.0]
**Gender**					
**Female**	0.97 [0.94–1.00]	0.02	1.01 [0.99–1.04]	0.37	0.6 [0.0–1.1]
**Ethnicity**					
**Non-Papuan**	Reference		Reference		
**Highland Papuan**	4.15 [3.92–4.40]	<0.001	3.80 [3.58–4.03]	<0.001	60.9 [60.1–61.6]
**Lowland Papuan**	1.45 [1.34–1.56]	<0.001	1.38 [1.28–1.49]	<0.001	1.8 [1.6–2.0]
**Year** a					
**2004**	Reference		Reference		
**2005**	1.28 [1.20–1.35]	<0.001	1.28 [1.21–1.36]	<0.001	
**2006**	1.22 [1.15–1.30]	<0.001	1.22 [1.15–1.30]	<0.001	
**2007**	1.61 [1.52–1.71]	<0.001	1.61 [1.52–1.71]	<0.001	
**2008**	1.58 [1.49–1.68]	<0.001	1.61 [1.52–1.71]	<0.001	
**2009**	1.91 [1.80–2.02]	<0.001	1.79 [1.69–1.90]	<0.001	
**2010**	1.78 [1.67–1.88]	<0.001	1.65 [1.56–1.75]	<0.001	
**2011**	1.46 [1.38–1.55]	<0.001	1.39 [1.31–1.48]	<0.001	
**2012**	1.99 [1.88–2.11]	<0.001	1.92 [1.82–2.04]	<0.001	
**Department** b					
**Outpatient**	Reference				
**Inpatient**	0.72 [0.71–0.74]	<0.001			

^a^ Included in model for calculation of overall PAFs but PAFs not presented.

^b^ Not included in the multivariable model as deemed to be a consequence of severe disease rather than a confounder.

OR, odds ratio.

### Reduction in Hemoglobin Concentration Associated with Malaria

**Figure 3:**
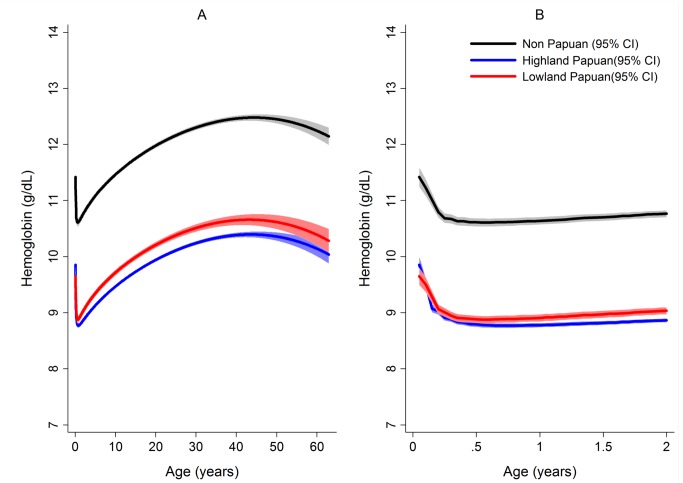
Estimated mean hemoglobin concentration in hospital attendees by ethnicity from infancy to adulthood (A) and during the first 2 years of life (B). Figures generated by multiple fractional polynomial regression analyses with the following covariables: ethnic group by age, *Plasmodium* species, sex, and year. Bands represent 95% CIs.

The mean hemoglobin concentration and prevalence of severe anemia varied significantly by *Plasmodium* species (Table 1). Mixed infection and *P. malariae* infection were associated with particularly poor hematological status (mean hemoglobin 9.22 g/dl and 8.93 g/dl, respectively; prevalence of severe anemia 8.3% and 5.9%, respectively) whilst the hematological statuses of patients infected with *P. vivax* and *P. falciparum* monoinfection were less severely impaired and broadly similar (Table 1). Highland Papuans had a mean hemoglobin of 9.78 g/dl compared to a mean of 10.09 g/dl in Lowland Papuans and 12.2 g/dl in non-Papuans; *p* for comparisons with non-Papuans <0.001 (Figure 3). Adult females had significantly lower mean hemoglobin concentrations compared to adult males (mean difference = −1.62 g/dl), and this remained apparent after excluding women known to be pregnant (mean difference = −1.46 g/dl); *p*<0.001.

**Figure 4:**
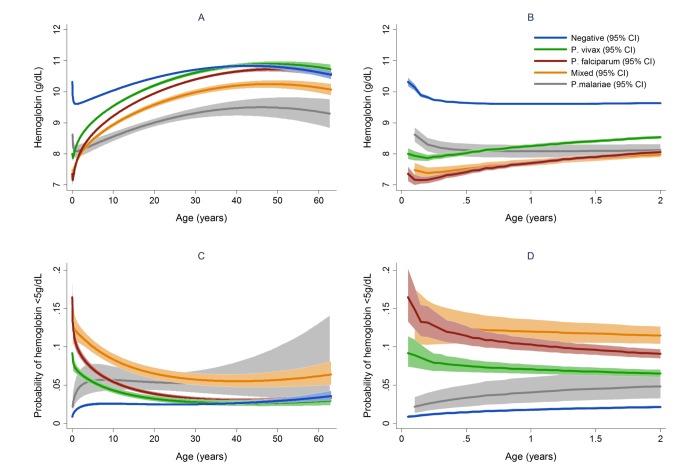
Estimated mean hemoglobin in hospital attendees by *Plasmodium* species from infancy to adulthood (A) and during the first 2 years of life (B) and the estimated probability of severe anemia (hemoglobin <5 g/dl) by *Plasmodium* species from infancy to adulthood (C) and during the first 2 years of life (D). Figures generated by multiple fractional polynomial regression analyses with the following covariables: *Plasmodium* species by age, sex, ethnic group, and year. Bands represent 95% CIs.

**Figure 5:**
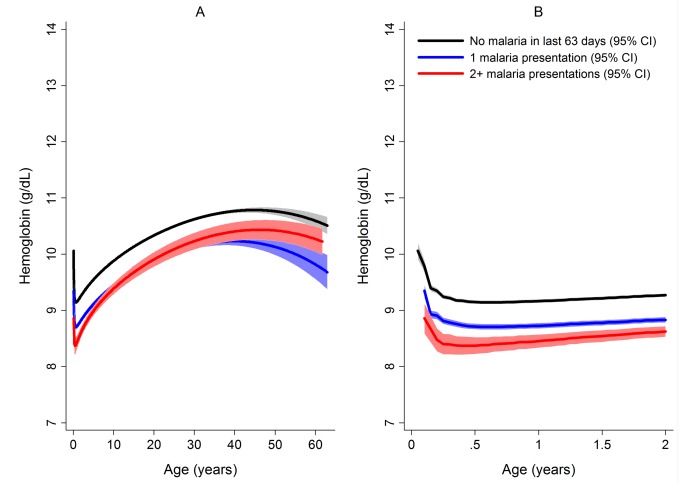
Estimated mean hemoglobin in hospital attendees by number of malaria episodes in the last 63 days from infancy to adulthood (A) and during the first 2 years of life (B). 189,671 patients had no recent episodes of malaria, 30,174 had one episode, and 4,572 had two or more episodes. Figures were generated by multiple fractional polynomial regression analysis with the following covariables: number of previous malaria episodes by age, *Plasmodium* species, ethnic group, sex, and year. Bands represent 95% CIs.

After correcting for confounding factors, patients presenting to hospital with malaria had lower mean hemoglobin concentrations and higher odds of severe anemia than patients without malaria at all ages, but most noticeably during childhood (Figure 4). Overall, *P. malariae* was associated with the greatest difference in mean hemoglobin compared to those without malaria (−1.40 g/dl [95% CI −1.52 to −1.29 g/dl]) followed by mixed infection (−1.01 g/dl [95% CI −1.07 to −0.95 g/dl]), *P. falciparum* (−0.70 g/dl [95% CI −0.73 to −0.66 g/dl]), and *P. vivax* (−0.58 g/dl [95% CI −0.62 to −0.54 g/dl]), *p* for all comparisons <0.001. Patients without a presentation to hospital with malaria within the preceding 2 months had a mean hemoglobin of 10.32 g/dl (95% CI 10.30–10.33 g/dl) compared to 9.42 g/dl (95% CI 9.39–9.45 g/dl) in patients with a single recent episode of malaria and 8.90 g/dl (95% CI 8.83–8.97 g/dl) in those with two or more episodes of malaria; *p*<0.001 (Figure 5; Table 1).

### The Risk of Severe Anemia

In total 97,665 (44.4%) of the 219,845 patient presentations were associated with a hemoglobin concentration less than 10 g/dl, 27,106 (12.3%) less than 7 g/dl, and 8,151 (3.7%) less than 5 g/dl. Compared to patients without malaria, those with mixed *Plasmodium* species infections were at the greatest risk of severe anemia (adjusted odds ratio [AOR] 3.25 [95% CI 2.99–3.54]) followed by those with *P. malariae* (AOR 2.18 [95% CI 1.76–2.67]), *P. falciparum* (AOR 2.11 [95% CI 2.00–2.23]), and *P. vivax* (AOR 1.87 [95% CI 1.74–2.01]), *p* for all comparisons <0.001 (Table 3). Patients with a recent presentation to hospital with *P. falciparum* malaria were at greater risk of severe anemia compared to those with recent *P. vivax* infection (5.3% [783/14,743] versus to 3.7% [459/12,507]; AOR = 1.51 [95% CI 1.32–1.73]; p<0.001). The comparative reduction in hemoglobin associated with *P. malariae* was greatest in adulthood whereas for *P. falciparum*, *P. vivax*, and mixed infections, it was worst during infancy (Figure 4).

**Table 3:** Risk factors for severe anemia (hemoglobin <5 g/dl).

Characteristics	Univariable Analysis		Multivariable Analysis		
	Crude OR [95% CI]	*p*-Value	AOR [95% CI]	*p*-Value	Overall PAF % [95% CI]
**Species**					
**Negative**	Reference		Reference		
***P. falciparum***	2.69 [2.54–2.84]	<0.001	2.12 [2.00–2.25]	<0.001	15.17 [13.96–16.37]
***P. vivax***	2.15 [2.00–2.32]	<0.001	1.88 [1.75–2.02]	<0.001	5.79 [5.01–6.57]
***P. malariae***	2.42 [1.95–3.01]	<0.001	2.18 [1.75–2.71]	<0.001	0.61 [0.38–0.84]
**Mixed species**	3.49 [3.20–3.81]	<0.001	3.27 [2.99–3.57]	<0.001	5.90 [5.29–6.50]
**Age group**					
**≥15 years**	Reference		Reference		
**<1 year**	1.01 [0.91–1.11]	0.89	1.05 [0.95–1.16]	0.34	0.35 [−0.29 to 0.99]
**1 to <5 years**	1.63 [1.52–1.75]	<0.001	1.42 [1.33–1.52]	<0.001	7.20 [5.99–8.40]
**5 to <15 years**	1.50 [1.38–1.63]	<0.001	1.24 [1.14–1.35]	<0.001	2.74 [1.84–3.63]
**Gender**					
**Female**	1.04 [0.98–1.10]	0.21	1.14 [1.07–1.20]	<0.001	6.25 [4.00–8.45]
**Ethnicity**					
**Non-Papuan**	Reference		Reference		
**Highland Papuan**	4.13 [3.66–4.66]	<0.001	3.66 [3.24–4.14]	<0.001	60.65 [57.63–63.45]
**Lowland Papuan**	2.52 [2.18–2.91]	<0.001	2.39 [2.07–2.77]	<0.001	5.97 [5.15–6.79]
**Year** a					
**2004**	Reference		Reference		
**2005**	0.60 [0.54–0.66]	<0.001	0.57 [0.51–0.63]	<0.001	
**2006**	0.68 [0.61–0.75]	<0.001	0.71 [0.64–0.78]	<0.001	
**2007**	0.89 [0.81–0.98]	<0.001	0.94 [0.85–1.03]	0.18	
**2008**	0.60 [0.54–0.67]	<0.001	0.68 [0.61–0.75]	<0.001	
**2009**	0.36 [0.33–0.41]	<0.001	0.36 [0.33–0.41]	<0.001	
**2010**	0.31 [0.28–0.35]	<0.001	0.31 [0.28–0.35]	<0.001	
**2011**	0.28 [0.25–0.32]	<0.001	0.29 [0.26–0.33]	<0.001	
**2012**	0.29 [0.26–0.33]	<0.001	0.29 [0.26–0.32]	<0.001	
**Department** b					
**Outpatient**	Reference				
**Inpatient**	6.79 [6.44–7.16]	<0.001			
**Presentations with malaria in the last 2 months** c					
**No episodes**	Reference				
**1 episode**	1.22 [1.14–1.31]	<0.001			
**2 or more episodes**	1.57 [1.36–1.82]	<0.001			

^a^ Included in model for calculation of overall PAFs but PAFs not presented.

^b^ Not included in the multivariable model as admission was deemed to be a consequence of severe disease rather than a confounder.

^c^ Number of previous presentations not included in the multivariable model as this would potentially obscure the net hematological effect of *Plasmodium* infection.

OR, odds ratio.

**Figure 6:**
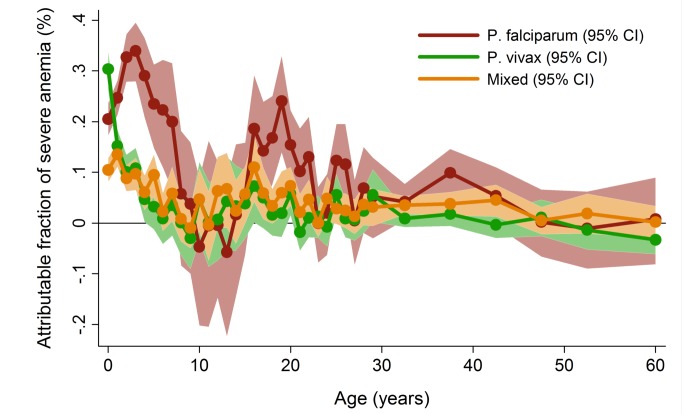
Adjusted population attributable fractions of severe anemia (hemoglobin <5 g/dl) by *Plasmodium* species and age. Figure generated by multiple logistic regression analyses with the following covariables for each age stratum: *Plasmodium* species, sex, ethnic group, and year. Bands represent 95% CIs.

In patients presenting to hospital the overall adjusted population fraction of severe anemia attributable to *P. falciparum* was 15.1% (95% CI 13.9–16.3%), compared to 5.75% (4.97%–6.53%) for *P. vivax*, 0.61% (0.38%–0.84%) for *P. malariae*, and 5.89% (5.28%–6.50%) for mixed species infections. These attributable fractions were unchanged when pregnant women were excluded. The fraction of severe anemia attributable to *P. vivax, P. malariae*, or mixed species infections was 12.2% (95% CI 11.2%–13.3%). The fraction of severe anemia attributable to *P. vivax* was highest in infancy, when it rose to 30.4% (95% CI 26.4%–34.2%) compared to 20.5% (95% CI 17.2%–23.7%) for *P. falciparum*. Following childhood, a second peak in the fraction of anemia attributable to malaria was apparent for all species between the ages of approximately 15 and 25 years (Figure 6).

### The Consequences of Severe Anemia

Admission for inpatient care was required in 63.6% (3,166/4,975) of the patients presenting initially to the outpatient department with severe anemia compared to 14% (21,481/151,454) of those without severe anemia (AOR = 6.34 [95% CI 6.00–6.69], *p*<0.001) (Table 4). Inpatients with severe anemia had a longer hospital stay (median 4 days [interquartile range (IQR) 2–7 days]) compared to 3 days (IQR = 2–4 days) in inpatients without severe anemia (*p*<0.001).

**Table 4:** Univariable and multivariable analyses of the risk factors for admission to hospital from the outpatient department (*n* = 156,429).

Characteristics	Univariable Analysis		Multivariable Analysis		
	Crude OR [95% CI]	*p*-Value	AOR [95% CI]	*p*-Value	PAF % [95% CI]
**Anemia**					
**Hemoglobin ≥5 g/dl**	Reference		Reference		
**Hemoglobin <5 g/dl**	6.79 [6.44–7.16]	<0.001	6.34 [6.00–6.69]	<0.001	4.9 [4.8–5.0]
**Species**					
**Negative**	Reference		Reference		
***P. falciparum***	1.66 [1.62–1.70]	<0.001	1.62 [1.58–1.67]	<0.001	5.6 [5.3–5.9]
***P. vivax***	0.84 [0.81–0.87]	<0.001	0.86 [0.83–0.89]	<0.001	<0 [<0–<0]
***P. malariae***	0.76 [0.68–0.85]	<0.001	0.73 [0.65–0.83]	<0.001	<0 [<0–<0]
**Mixed species**	1.38 [1.32–1.44]	<0.001	1.50 [1.43–1.57]	<0.001	1.0 [0.9–1.1]
**Age group**					
**≥15 years**	Reference		Reference		
**<1 year**	1.02 [0.99–1.06]	0.16	1.14 [1.10–1.18]	<0.001	0.8 [0.6–1.0]
**1 to <5 years**	0.77 [0.75–0.79]	<0.001	0.76 [0.73–0.78]	<0.001	<0 [<0–<0]
**5 to <15 years**	0.64 [0.62–0.66]	<0.001	0.59 [0.57–0.61]	<0.001	<0 [<0–<0]
**Gender**					
**Female**	1.21 [1.18–1.24]	<0.001	1.19 [1.17–1.22]	<0.001	6.2 [5.6–6.9]
**Ethnicity**					
**Non-Papuan**	Reference		Reference		
**Highland Papuan**	1.11 [1.08–1.15]	<0.001	1.10 [1.06–1.13]	<0.001	4.1 [2.9–5.3]
**Lowland Papuan**	1.35 [1.29–1.40]	<0.001	1.37 [1.31–1.43]	<0.001	2.9 [2.6–3.2]
**Year** a					
**2004**	Reference		Reference		
**2005**	0.94 [0.90–0.99]	0.01	1.00 [0.96–1.05]	0.98	
**2006**	1.20 [1.15–1.26]	<0.001	1.30 [1.24–1.36]	<0.001	
**2007**	1.22 [1.17–1.28]	<0.001	1.27 [1.21–1.33]	<0.001	
**2008**	1.24 [1.18–1.30]	<0.001	1.37 [1.31–1.44]	<0.001	
**2009**	0.75 [0.72–0.78]	<0.001	0.86 [0.82–0.90]	<0.001	
**2010**	0.63 [0.60–0.66]	<0.001	0.73 [0.69–0.76]	<0.001	
**2011**	0.66 [0.63–0.70]	<0.001	0.77 [0.73–0.81]	<0.001	
**2012**	0.54 [0.51–0.56]	<0.001	0.61 [0.58–0.64]	<0.001	
**Presentation with malaria in the last 2 months** b					
**No episodes**	Reference				
**1 episode**	0.75 [0.73–0.78]	<0.001			
**2 or more episodes**	0.87 [0.81–0.93]	<0.001			

^a^ Included in model for calculation of overall PAFs but PAFs not presented.

^b^ Number of previous presentations not included in the multivariable model as this would potentially obscure the net clinical severity of *Plasmodium* infection.

OR, odds ratio.

**Figure 7:**
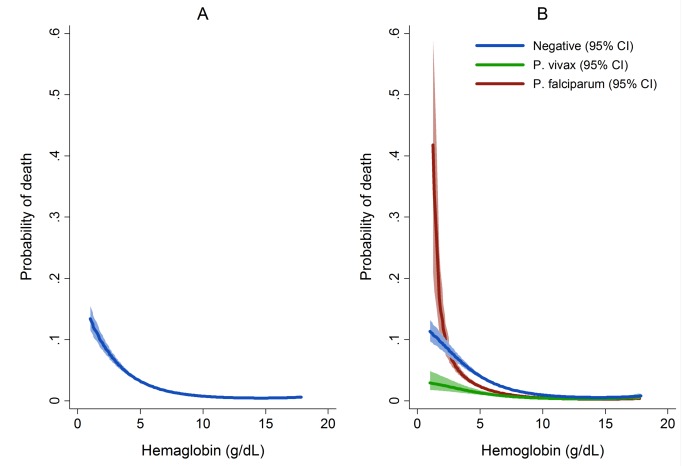
Estimated probability of mortality by hemoglobin concentration overall (A) and by *Plasmodium* species (B). Figures generated by multiple fractional polynomial regression analyses with the following covariables: *Plasmodium* species, hemoglobin, age, sex, ethnic group, and year. Bands represent 95% CIs. Risk of death in Figure 6A = 1/(1+exp(−(−1.44−0.4146 * hb+0.0006 * hb^3^))), where the other covariates in the model are set equal to their mean value (for continuous variables) or prevalence (for indicator variables).

The overall risk of mortality in those admitted to hospital with a hemoglobin measurement was 1.3% (2,781/219,845), the risk increasing as the hemoglobin concentration fell below 7 g/dl (Figure 7). In total 1.5% (2,254/152,149) of patients without malaria died compared to 0.9% (340/37,554) with *P. falciparum* infection, 0.6% (112/19,858) with *P. vivax* infection, 0.9% (0.9% 14/1,608) with *P. malariae* infection, and 0.7% (61/8,645) with mixed infection. The odds ratio for death in patients with a hemoglobin concentration lower than 5 g/dl was 4.63 (95% CI 4.16–5.16), compared to those with a higher hemoglobin concentration, *p*<0.001. After controlling for age, the AOR of death associated with severe anemia was 5.91 (95% CI 5.17–6.76) for patients without concurrent malaria, 5.93 (95% CI 4.63–7.58) for those with *P. falciparum* infection, 5.33 (95% CI 3.10–9.17) for mixed infections, 4.43 (95% CI 2.76–7.11) for *P. vivax*, and 6.58 (95% CI 2.02–21.47) for those with *P. malariae*. The PAF of death associated with severe anaemia was 11.9% (95% CI 10.6%–13.2%). The full multivariable model for mortality is presented in Table 5.

**Table 5:** Univariable and multivariable analyses of the risk factors for death (*n* = 219,845).

Characteristics	Univariable Analysis		Multivariable Analysis		
	Crude OR [95% CI]	*p*-Value	AOR [95% CI]	*p*-Value	PAF % [95% CI]
**Anemia**					
**Hemoglobin ≥5 g/dl**	Reference		Reference		
**Hemoglobin <5 g/dl**	4.63 [4.16–5.16]	<0.001	5.80 [5.17–6.50]	<0.001	11.9 [10.6–13.2]
**Species**					
**Negative**	Reference		Reference		
***P. falciparum***	0.61 [0.54–0.68]	<0.001	0.57 [0.51–0.65]	<0.001	<0 [<0–<0]
***P. vivax***	0.38 [0.31–0.46]	<0.001	0.37 [0.31–0.45]	<0.001	<0 [<0–<0]
***P. malariae***	0.58 [0.34–0.99]	0.05	0.57 [0.34–0.98]	0.04	<0 [<0–<0]
**Mixed species**	0.47 [0.37–0.61]	<0.001	0.45 [0.35–0.59]	<0.001	<0 [<0–<0]
**Age group**					
**≥15 years**	Reference		Reference		
**<1 year**	1.43 [1.28–1.60]	<0.001	1.40 [1.25–1.58]	<0.001	4.0 [2.5–5.4]
**1 to <5 years**	0.60 [0.53–0.67]	<0.001	0.62 [0.55–0.70]	<0.001	<0 [<0–<0]
**5 to <15 years**	0.57 [0.49–0.66]	<0.001	0.60 [0.51–0.69]	<0.001	<0 [<0–<0]
**Gender**					
**Female**	0.63 [0.58–0.68]	<0.001	0.58 [0.54–0.63]	<0.001	<0 [<0–<0]
**Ethnicity**					
**Non-Papuan**	Reference		Reference		
**Highland Papuan**	0.61 [0.55–0.67]	<0.001	0.64 [0.58–0.71]	<0.001	<0 [<0–<0]
**Lowland Papuan**	0.93 [0.83–1.05]	0.26	0.95 [0.84–1.08]	0.46	<0 [<0–1.4]
**Year** a					
**2004**	Reference		Reference		
**2005**	0.99 [0.81–1.21]	0.94	1.09 [0.89–1.33]	0.39	
**2006**	1.41 [1.16–1.71]	<0.001	1.47 [1.21–1.79]	<0.001	
**2007**	1.57 [1.30–1.89]	<0.001	1.54 [1.27–1.86]	<0.001	
**2008**	1.85 [1.53–2.24]	<0.001	1.84 [1.52–2.23]	<0.001	
**2009**	0.93 [0.77–1.13]	0.48	1.04 [0.85–1.27]	0.70	
**2010**	0.77 [0.63–0.94]	0.01	0.87 [0.71–1.07]	0.19	
**2011**	0.95 [0.78–1.16]	0.60	1.05 [0.86–1.29]	0.61	
**2012**	0.87 [0.71–1.05]	0.15	0.99 [0.81–1.20]	0.90	
**Presentation with malaria in the last 2 months** b					
**No episodes**	Reference				
**1 episode**	0.63 [0.54–0.72]	<0.001			
**2 or more episodes**	0.59 [0.42–0.82]	<0.001			

^a^ Included in model for calculation of overall PAFs but PAFs not presented.

^b^ Number of previous presentations not included in the multivariable model as this would potentially obscure the net clinical severity of *Plasmodium* infection.

OR odds ratio.

## Discussion

Anemia is highly prevalent throughout the tropics and has major consequences for human health and socioeconomic prosperity [40]. Our large hospital-based study in southern Papua, Indonesia provides a comparative assessment of the pattern of hematological changes associated with the non-falciparum malarias as well as an estimate of their public health importance. We have shown that *P. vivax* is associated with a high burden of severe anemia in infancy (almost one-quarter of all cases at the hospital), that mixed *P. vivax/P. falciparum* infection causes significantly more hematological impairment than monoinfection with either species alone, and that in adulthood *P. malariae* infection is associated with the lowest mean hemoglobin concentration. Approximately 12% of severe anemia at the hospital can be attributed to non-falciparum *Plasmodium* infection, the consequences of which include a 6-fold greater risk of admission to hospital and an increased duration of inpatient stay. More importantly, lower hemoglobin concentrations were associated with a 5-fold increased risk of death, the worst prognosis being in patients with severe anemia associated with falciparum malaria and those without concurrent malaria.

Infancy is a time of rapid physical and cognitive development as well as increased vulnerability to infectious diseases. Our study shows that the risk of symptomatic vivax malaria is more heavily skewed towards infancy compared with other *Plasmodium* species, probably reflecting more efficient transmission and faster acquisition of immunity. For a given parasite density, *P. vivax* evokes a greater immune response [41],[42] than other species. Subsequent parasite exposure is also higher due to its propensity to relapse multiple times. In our study, patients with *P. vivax* infection were significantly more likely to have had a prior presentation with malaria in the preceding 2 months compared with the other species. Infants are particularly vulnerable to recurrent episodes of vivax malaria, a likely consequence of lower immunity and greater inherent susceptibility to infection; *P. vivax* has a strong predilection for reticulocytes, which are most abundant in the first 3 months of life [43].

Our study confirms *P. vivax* as the predominant malaria infection in infancy and highlights its frequent association with severe anemia in this age group. Young children with severe falciparum malarial anemia have previously been shown to have an increased risk of mortality [5],[11],[40] and blood transfusion (with attendant risk of blood borne disease transmission) [5] and they may also have reduced resilience to other infectious and non-infectious diseases. In our study, patients with vivax malaria and severe anemia had a 4.4-fold increase in the probability of death from any cause compared to those without severe anaemia. However the risk of death in those with severe anemia without concomitant malaria and, in particular, severe anemia caused by *P. falciparum* infection was much higher (Figure 5). It is possible that this is due to *P. falciparum* being associated with higher parasitemia and potentially a faster and larger decrement in hemoglobin; a rapid drop in hemoglobin permitting less time for the body to make physiological adjustments to ameliorate deleterious effects of severe anemia. In contrast severe anemia caused by *P. vivax* infection is more likely to be due to a chronic process, resulting from the accumulation of smaller hemoglobin decrements with each recurrent bout of infection. This hypothesis is supported by the greater reduction within a single clinical encounter (maximum to minimum hemoglobin concentration) in patients with *P. falciparum* (0.21 g/dl) and mixed infections (0.24 g/dl) compared to those with *P. vivax* monoinfection (0.17 g/dl), and the 1.5-fold greater risk of severe anemia observed following recent falciparum malaria compared to those with recent vivax malaria. In non-malarial severe anemia the greater mortality may reflect a more acute onset compounded by a greater number or severity of comorbidities. Our study was unable to assess the long term effects of malarial anemia, which in other studies has been associated with adverse developmental and socioeconomic sequelae, although defining these is challenging due to the multifactorial aetiology of anemia in malaria-endemic areas [4],[11],[40],[44]. Malaria has been linked with impaired cognitive development, but it is unclear if this is due to the hematological effects of infection or the infection itself [44].

Previous studies in Thailand have shown an association between concomitant *P. vivax* infection and reduced severity of *P. falciparum* infection [30],[31]. However this was not apparent in southern Papua where mixed infections after 3 years of age were associated with both a lower mean hemoglobin concentration and greater odds of severe anemia than monoinfection with either species. This could reflect greater antigenic diversity of local parasite strains and thus less cross-reactive immunity, though empiric evidence for this is lacking. Alternatively, it may relate to greater transmission intensity in southern Papua. In this region the majority of severe malarial anemia beyond childhood is likely to be the result of repetitive infections in individuals who have already developed partial immunity. In this situation, the hematological effects of the two species are likely to be additive and any immunomodulatory effect of *P. vivax* on *P. falciparum* is likely to be negligible. In Thailand, where transmission is less intense, severe falciparum anemia is more likely to result from a single fulminant infection in a non-immune individual and therefore any immunomodulatory effect of *P. vivax* would be proportionately more important.

Somewhat unexpectedly, *P. malariae* was found to be associated with the lowest mean hemoglobin of all locally prevalent *Plasmodium* species, and a high frequency of severe anemia. Its hematological effects were most apparent in adulthood whereas in childhood it was associated with minimal impairment. *Plasmodium. malariae* is a slow-growing parasite that usually causes a low parasitemia [45]. It is believed to have a predilection for old red blood cells [45] and hence should have a minimal effect on the average lifespan of circulating red blood cells [46]. A high proportion of *P. malariae* infections is asymptomatic and therefore unlikely to prompt presentation to a health care service [37]. We suspect that the severity of the hematological impairment seen in this study is related to prolonged red cell destruction and bone marrow suppression as a result of chronic, asymptomatic, or minimally symptomatic parasitemia. *Plasmodium malariae* is prevalent in much of sub-Saharan Africa [47] and according to the results of this study may be associated with an underappreciated burden of anemia in adults.

Our study has several strengths. It includes patients with uncomplicated malaria presenting to the outpatients department through to those with complicated disease who were admitted to hospital. The analysis was focused on clinical events, with each patient presenting in some cases multiple times over the 8 years of the study period. All children greater than 1 week of age were included whereas other studies have excluded those under 6 months of age [26]. Cases of malaria were microscopically confirmed by quality-assured microscopists and data entry was carried out in a consistent fashion. Because of strict hospital policy, the vast majority of symptomatic malaria infections were likely to have been ascertained. There are also a number of limitations to our study. Hemoglobin measurement was done on an as-needed basis and therefore a degree of selection bias has almost certainly occurred. Although the majority of patients with malaria did not have a hemoglobin measurement, the proportion of patients who did have a measurement was the same for all species (*P. ovale* excluded) and hence, comparisons of the hematological effects of the different species are likely to be valid. Those without malaria were less likely to have a full blood count, both in the outpatient and inpatient setting. However, the low threshold for hematological investigation at the hospital suggests that patients who did not have a blood test would be unlikely to have severe anemia. Furthermore the absence of malaria at the time of the clinical encounter does not preclude the contribution to anemia of prior, recently treated, asymptomatic, or submicroscopic parasitemia. Our study was also limited to observations documented during the hospital encounter and could not address out-of-hospital deaths associated with severe anaemia. However these biases are likely to have resulted in the derived malaria-attributable fractions of severe anemia and their associated morbidity and mortality being conservative, if anything underestimating the true burden of disease.

Our analyses may have also been subject to residual confounding. Hemoglobin and red cell polymorphisms are known to influence the likelihood of severe malarial anemia [48]–[50] and in some cases may also modulate the risk of uncomplicated parasitemia [51]–[56]. We adjusted for ethnicity, which, in Papua, is strongly indicative of the geographic location and altitude of tribal lands and thus should have accounted for at least some of the potential variation in the prevalence of these disorders by malaria status.

Other potential confounders include nutritional status, geohelminth infestation, bacteremia, chronic disease, and pregnancy status. Apart from pregnancy status, information on these confounders was not available. Malaria and malnutrition may cluster in the same geographic areas, but the mechanisms and direction of any biological link have not been elucidated conclusively. Chronic vivax malaria has been implicated in a state of malnourishment akin to kwashiorkor [57], whereas iron deficiency may provide some protection against malaria infection [58]. Geohelminth infection causes gastrointestinal blood loss, which may exacerbate anemia caused by *P. falciparum* malaria [59]. Two oft-cited studies by Nacher and Spiegel, respectively, indicate that intestinal helminthiasis increases the risk of falciparum malaria by a factor of 1.5 and 2.2 [60],[61]. The relationship between geohelminth infection and *P. vivax* malaria is unclear [62],[63]. There is a potential that the severity of anemia, and hence the attributable fraction of anemia, associated with *Plasmodium* infection in our study may have been overestimated by our inability to control for geohelminth infection. This is unlikely to have been important for children under 3 years old since helminth density typically peaks in adulthood and previous work has shown minimal impact on hemoglobin concentrations prior to 30 months of age [64].

Pregnancy may have also confounded the association of anaemia and malaria in adults. Pregnant women are at comparatively high risk of *P. falciparum*, and possibly *P. vivax*, parasitemia in malaria endemic regions [65],[66]. Our previous studies at the same hospital in southern Papua have demonstrated that primigravidae women are at increased risk of severe malarial anemia [36]. Reassuringly in a subgroup analysis the exclusion of women known to be pregnant did not alter the PAF of anemia associated with malaria.

Finally the results of this study cannot necessarily be generalised to individuals with asymptomatic disease. Although cross-sectional survey data could be used to compare the hematological impact of symptomatic and asymptomatic *Plasmodium* infection this approach would tend to miss patients at the severe end of the spectrum.

In conclusion our very large hospital-based study demonstrates that the non-falciparum malarias make a major contribution to the burden of anemia in southern Papua, with 12% of all severe anemia at the hospital attributable to *P. vivax, P. malariae*, or mixed species infections. *Plasmodium vivax* was associated with the greatest attributable fraction of severe anemia in infancy and is likely to be an important cause of indirect morbidity. Conversely, the hematological impact of *P. malariae* was most apparent in adults. In contrast to earlier reports from regions of low mixed-species endemicity, mixed *P. falciparum/P. vivax* infections are associated with a significantly greater absolute reduction in hemoglobin and odds of severe anemia than monoinfection with either species alone. Severe malarial anemia, including that caused by *P. vivax* infection, is associated with an increased likelihood of admission, a prolonged hospital stay, and, particularly with *P. falciparum* infections, dramatically increased mortality. These findings highlight the public health importance of integrated genus-wide rather than species-specific malaria control strategies in areas of *Plasmodium* co-endemicity.

## Abbreviations

PAF: population attributable fraction
SD: standard deviation
